# PPAR**α**-Independent Arterial Smooth Muscle Relaxant Effects of PPAR**α** Agonists

**DOI:** 10.1155/2012/302495

**Published:** 2012-09-11

**Authors:** Neerupma Silswal, Nikhil K. Parelkar, Michael J. Wacker, Mostafa Badr, Jon Andresen

**Affiliations:** ^1^Muscle Biology Research Group (MUBIG), Basic Medical Science Department, School of Medicine, University of Missouri-Kansas City, Kansas City, MO 64108, USA; ^2^Division of Pharmacology and Toxicology, School of Pharmacy, University of Missouri-Kansas City, Kansas City, MO 64108, USA

## Abstract

We sought to determine direct vascular effects of peroxisome proliferator-activated receptor alpha (PPAR**α**) agonists using isolated mouse aortas and middle cerebral arteries (MCAs). The PPAR**α** agonists GW7647, WY14643, and gemfibrozil acutely relaxed aortas held under isometric tension and dilated pressurized MCAs with the following order of potency: GW7647≫WY14643>gemfibrozil. Responses were endothelium-independent, and the use of PPAR**α** deficient mice demonstrated that responses were also PPAR**α**-independent. Pretreating arteries with high extracellular K^+^ attenuated PPAR**α** agonist-mediated relaxations in the aorta, but not in the MCA. In the aorta, the ATP sensitive potassium (K_ATP_) channel blocker glibenclamide also impaired relaxations whereas the other K^+^ channel inhibitors, 4-aminopyridine and Iberiotoxin, had no effect. In aortas, GW7647 and WY14643 elevated cGMP levels by stimulating soluble guanylyl cyclase (sGC), and inhibition of sGC with ODQ blunted relaxations to PPAR**α** agonists. In the MCA, dilations were inhibited by the protein kinase C (PKC) activator, phorbol 12,13-dibutyrate, and also by ODQ. Our results demonstrated acute, nonreceptor-mediated relaxant effects of PPAR**α** agonists on smooth muscle of mouse arteries. Responses to PPAR**α** agonists in the aorta involved K_ATP_ channels and sGC, whereas in the MCA the PKC and sGC pathways also appeared to contribute to the response.

## 1. Introduction

Peroxisome proliferator-activated receptors (PPARs), of which there are three subtypes (*α*, *γ*, and *β*/*δ*), are clinically important pharmacological targets for the treatment of diabetes, dyslipidemia, and metabolic syndrome [1]. First to be cloned, PPAR*α* is widely expressed in liver, heart, skeletal muscle, brown adipose, endothelium, and vascular smooth muscle [2–8]. Biologic PPAR*α* agonists consist of saturated and unsaturated fatty acids, eicosanoids, and glucocorticoids [9–15]. Synthetic PPAR*α* agonists include herbicides, plasticizers, fibrates, WY14643, and GW7647. Fibrates are in clinical use and have cardioprotective effects including reduced death from coronary heart disease, and prevention of myocardial infarction [16–21]. In addition, fibrates reduce stroke occurrence [22], decrease atherosclerosis [23], suppress inflammatory responses in vascular smooth muscle cells [7, 24, 25], and enhance nitric oxide (NO•) production in endothelial cells [26]. Although not in clinical use, GW7647 prevents atherosclerosis in hyperlipidemic mice [27], and WY14643 suppresses the inflammatory response in human aortic smooth muscle cells [7]. Thus, PPAR*α* agonists appear to protect the cardiovascular system from inflammation and disease.

Although PPAR*α* agonists positively impact cardiovascular outcomes, their effects specifically in the vasculature are less well understood. Treating mice with fenofibrate for ten days enhanced endothelium-dependent dilation of resistance (mesenteric) and large conduit (aorta) arteries, possibly by increasing responsiveness to NO• [28]. Likewise, fourteen days in vivo treatment with fenofibrate modestly improved endothelium-dependent dilation of the mouse middle cerebral artery (MCA) [29]. Feeding low levels of WY14643 to mice over ten days also resulted in reduced systolic pressure [30]. Emerging evidence suggests that PPAR*α* agonists also have acute, possibly nonreceptor-mediated, effects such as visceral analgesia [31], increased insulin-induced glucose uptake [32], and stimulation of mitogen-activated protein kinases [33–35]. In the cardiovascular system, another PPAR*α* agonist, gemfibrozil, acutely lowered systemic arterial pressure, and directly relaxed tail arteries of rats by an undefined smooth muscle-dependent mechanism [36]. The fibrate compounds gemfibrozil, fenofibric acid, and bezafibrate also relaxed the rat thoracic aorta evidently by decreasing intracellular calcium, albeit at relatively high concentrations [37]. Therefore, PPAR*α* agonists appear to have both long-term (genomic and possibly nongenomic), as well as short-term (probably nongenomic and possibly nonreceptor-mediated) beneficial effects for the cardiovascular system. Thus, we aimed to determine direct effects of PPAR*α* agonists on isolated arteries and to delineate the mechanism by which they cause arterial relaxations.

Based on previous results, we predicted that PPAR*α* agonists would promote arterial relaxation. Using isometric tension and isobaric myography we examined the ability of the PPAR*α* agonists gemfibrozil, WY14643, and GW7647 to acutely relax the mouse aorta and to dilate the MCA. In addition, we sought to define the mechanism of action of the observed relaxant effect by using different pharmacological inhibitors and PPAR*α*-deficient (Ppar*α* ^−/−^) mice. Our results demonstrate that PPAR*α* agonists caused relaxation of mouse aorta by activating soluble guanylyl cyclase (sGC) and ATP sensitive potassium (K_ATP_) channels. The dilatory response in the MCA, however, involved activation of sGC as well as inhibition of protein kinase C (PKC).

## 2. Materials and Methods

### 2.1. Animals and Reagents

Male C57BL/6J and PPAR*α*-deficient (Ppar*α* ^−/−^) mice (aged 12–16 weeks) were purchased from The Jackson Laboratory (Bar Harbor, ME, USA). Mice were euthanized by CO_2_ inhalation and decapitated prior to tissue harvesting. Deletion of the PPAR*α* gene was confirmed by using primers available from the Jackson Laboratory database using standard PCR conditions. The Animal Care and Use Committee at the University of Missouri-Kansas City approved all protocols. All reagents were sourced from Sigma (St. Louis, MO, USA) unless otherwise noted. Stock solutions of PPAR*α* agonists were prepared in DMSO and diluted in Krebs buffer prior to use. Concentrations of DMSO in the bath never exceeded 0.01% and controls were always vehicle treated.

### 2.2. Isobaric Vessel Studies: MCA

Brains were quickly removed and placed in ice-cold Hank's buffered saline solution (HBSS, Invitrogen, Carlsbad, CA, USA). MCAs were studied in a pressurized artery myograph (DMT-USA, Ann Arbor, MI, USA) as previously described [38–41]. Briefly, MCAs were carefully dissected away from the brain, cleared of blood and pia mater, mounted on glass micropipettes, and pressurized to 70 mm Hg with Krebs buffer (in mM: 119 NaCl, 4.7 KCl, 0.24 NaHCO_3_, 1.18 KH_2_PO_4_, 1.19 MgSO_4_, 5.5 glucose, and 1.6 CaCl_2_). Elevated external K^+^ buffers were made isotonic by replacement of NaCl with KCl on an equimolar basis. Prior to beginning experiments smooth muscle function was verified with 60 mM KCl-induced contractions whereas endothelial function in phenylephrine (PE, 10^−5^ M) preconstricted MCAs was probed by determining dilation to 10^−5^ M acetylcholine. MCA's were denuded by passage of 1 mL of air, over a period of 8 min at 60 mm Hg as previously described [38]. MCAs were preconstricted with 10^−5^ M PE prior to determining dilatory responses to PPAR*α* agonists.

### 2.3. Isometric Tension Myography: Aorta

The thoracic aorta was rapidly excised and placed in ice-cold HBSS where blood, fat, and excess connective tissues were carefully removed. Segments 3-4 mm in length were mounted on pins in chambers of a DMT 610 M wire myograph system (Danish Myo Technology A/S, Aarhus N, Denmark) containing Krebs buffer saturated at 37°C with a gas mixture containing 20% O_2_/5% CO_2_/75% N_2_ (Airgas Mid South Inc., Tulsa, OK, USA). Arterial rings were progressively stretched to 0.75 g equivalent force passive tension in 0.1 g steps and allowed to equilibrate for 45 minutes described previously [41]. Aortic rings were exposed to isotonic KCl (40 and 80 mM), and also to 10^−5 ^M Prostaglandin F2-alpha (PGF_2*α*_) followed by 10^−6^ *μ*M acetylcholine (ACh) to assess the quality of the preparation. Vessels were rinsed once with fresh Krebs every 15 min and several times after concentration-response curves. Dose-response curve to PPAR*α* agonists was determined after preconstriction of aortic segments with 10^−5^ M PGF_2*α*_. In pharmacological inhibition experiments, selective agents were added 15–30 minutes prior to preconstriction with PGF_2*α*_, and then relaxation response to PPAR*α* agonists was determined. For denudation, the forceps were inserted into the lumen, and the aortic ring was gently rolled over. This process was repeated 5 times. The effectiveness of endothelium removal was confirmed by the absence of relaxation by 10^−6^ *μ*M Ach in aortic rings precontracted with 10^−5^ M PGF_2*α*_. Force changes were recorded using an ADinstruments (Colorado Springs, CO, USA) PowerLab 4/30 and associated LabChart Pro software (v6.1) running on a standard Windows XP computer platform.

### 2.4. Determination of Cyclic Guanosine Monophosphate**  **(cGMP) Content

The thoracic aorta was excised and cleaned of fat and excess connective tissue in ice-cold HBSS. The aorta was cut into rings of 3 mm in length and treated with PDE inhibitor 3-isobutyl-1-methylxanthine (IBMX; 10^−1 ^M) in HBSS for 15 minutes. Aortic rings were then incubated with vehicle (0.1% DMSO), GW7647 (10^−5 ^M), WY14643 (10^−4 ^M), 1H-[1,2,4]oxadiazolo[4,3-a]quinoxalin-1-one (ODQ, 10^−5 ^M), SNP (10^−5 ^M), the combination of GW7647 with ODQ, and WY14643 with ODQ, or SNP and ODQ for 15 minutes. After incubation aortic rings were immediately frozen in liquid nitrogen and stored at −80°C. For cGMP extraction, rings were homogenized in five volumes of ice-cold 5% (w/v) trichloroacetic acid followed by centrifugation at 2000 ×g for 15 minutes at 4°C. The supernatant was recovered, extracted four times with five volumes of water-saturated diethyl ether, and the extracted samples were then evaporated to dryness. cGMP content in the extracted samples was then determined by using a commercially available cGMP EIA kit (Caymen Chemical, Ann Arbor, MI, USA) according to the manufacturer's instructions.

### 2.5. Statistics

Data are plotted and expressed as means ± SEM and *n*-values are detailed in the legends to the figures. In myograph experiments, changes in isometric tension are expressed as % relaxation. Changes in the diameter of pressurized and MCAs were calculated as % dilation as previously described [38]. Two-factor ANOVA was used to determine differences between concentration-response curves. One-factor ANOVA with Tukey's multiple-comparison *post hoc* tests were used cGMP assay data. Where appropriate, data normality was examined with D'Agostino and Pearson tests and homogeneity of variance was determined with Bartlett's test. Data were plotted and statistics computed with Graphpad Prism (v5.01, San Diego, CA, USA). Significance was accepted at *P* ≤ 0.05.

## 3. Results

### 3.1. Responses to PPAR*α* Agonists in Aortas and MCAs

**Figure 1:**
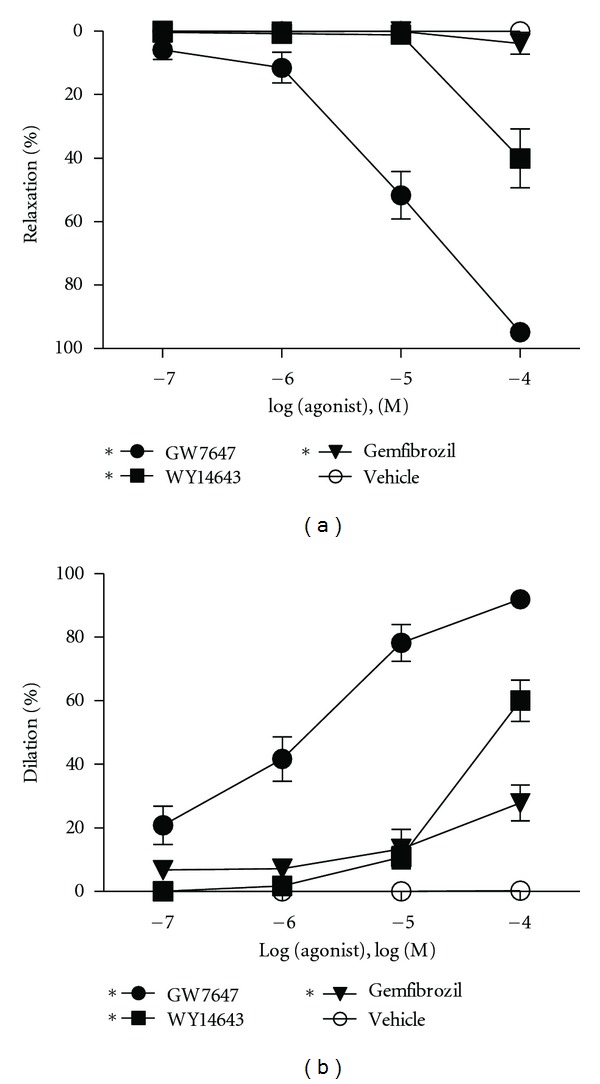
Response to PPAR*α* agonists in aortas and MCAs of WT mice. Concentration-response curves to three different PPAR*α* agonists (gemfibrozil, WY14643, and GW7647) in (a) mouse aortic rings precontracted with 10^−5^ M PGF_2*α*_ by using isometric tension myography and (b) MCAs preconstricted with 10^−5^ M PE by using isobaric myography. All three PPAR*α* agonists relaxed aortic rings (*n* = 3–5) as well as dilated the MCA (*n* = 7). Data are means ± SEM. *Indicates *P* ≤ 0.05.

Previous studies demonstrated that relatively nonselective PPAR*α* agonists such as gemfibrozil acutely caused vasodilation of rat tail arteries, and relaxation of rat thoracic aorta [36, 37]. To expand upon and investigate this further we determined the direct and acute effects of three PPAR*α* agonists, gemfibrozil, WY14643, and GW7647, on isolated mouse arteries. In binding assays these agonists range from moderately specific and selective for PPAR*α* (gemfibrozil) to very specific and selective (GW7647) [42]. Cumulative addition of each agonist (10^−7^ M to 10^−4^ M) to the bath caused concentration-dependent relaxation of precontracted aortas; however, the effectiveness varied greatly for each agonist (Figure 1(a)). For example, the relaxation caused by 10^−4^ M GW7647 was 95 ± 1% whereas the same concentration of WY14643 only caused 40 ± 9% relaxation. Even at 10^−4^ M, gemfibrozil had little to no effect causing just 4 ± 3% relaxation of the aorta. Similar to the aorta, at 10^−4^ M GW7647 maximally dilated the MCA, whereas dilation to 10^−4^ M WY14643 and gemfibrozil was 60 ± 11% and 28 ± 5%, respectively, (Figure 1(b)). Vehicle (DMSO) treatment did not have any effect on aortic tension or MCA dilation (Figures 1(a) and 1(b)).  Figure 1 also shows that the dilatory potency increased in parallel with selectivity for PPAR*α* such that the highly selective (in specialized assays) PPAR*α* agonist GW7647 was substantially more potent than WY14643 or gemfibrozil.

### 3.2. Role of PPAR*α* Receptors in Vascular Responses to PPAR*α* Agonists 

**Figure 2:**
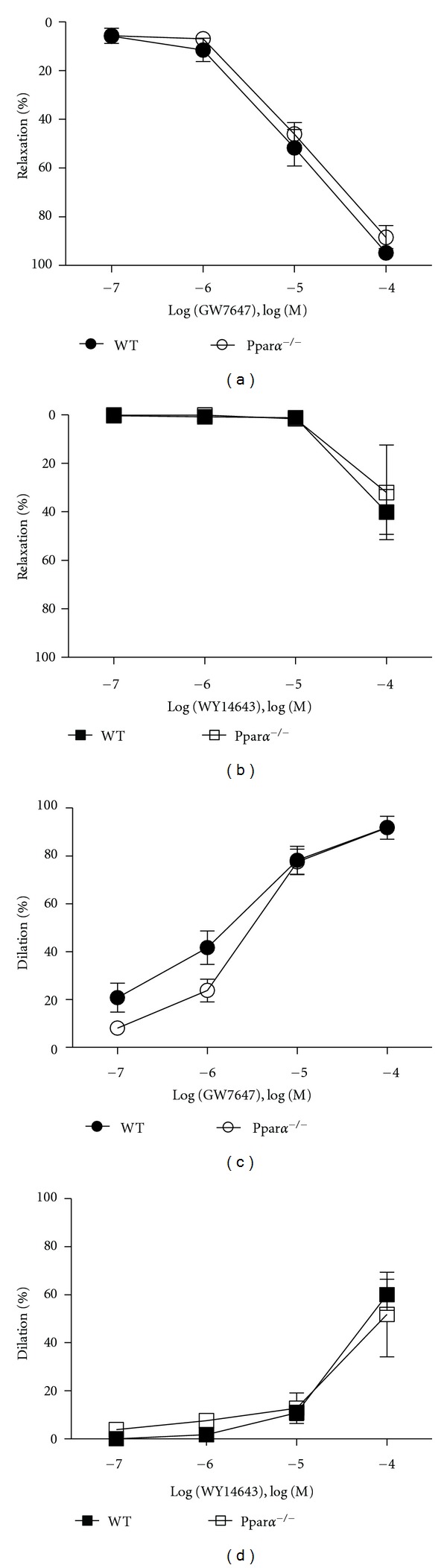
Nonreceptor-mediated responses to PPAR*α* agonists. Concentration responses to GW7647 and WY14643 in aortic rings (a) and (b) and in MCAs (c) and (d) isolated from WT and Ppar*α* ^−/−^ mice (*n* = 3–5). Responses to GW7647 and WY14643 in aortic rings and MCAs of Ppar*α* ^−/−^ mice were identical to that of WT (control) mice indicating a receptor-independent effect. Data are means ± SEM. *Indicates *P* ≤ 0.05.

To determine if the response caused by PPAR*α* agonists was receptor-mediated, we utilized Ppar*α* ^−/−^ mice that do not express PPAR*α*. Compared to WT mice, aortic responses were unaltered in Ppar*α* ^−/−^ mice for both GW7647 and WY14643 (Figures 2(a) and 2(b)). Likewise, as shown in Figures 2(c) and 2(d) dilation of Ppar*α* ^−/−^ MCAs did not differ from WT responses for either GW7647 or WY14643. Thus, these data indicated nonreceptor-mediated effects of PPAR*α* agonists on arteries. Therefore, we sought to determine the mechanism by which GW7647 and WY14643 mediated vasorelaxation.

### 3.3. Mechanism of Relaxation Caused by PPAR*α* Agonists in the Aorta 

**Figure 3:**
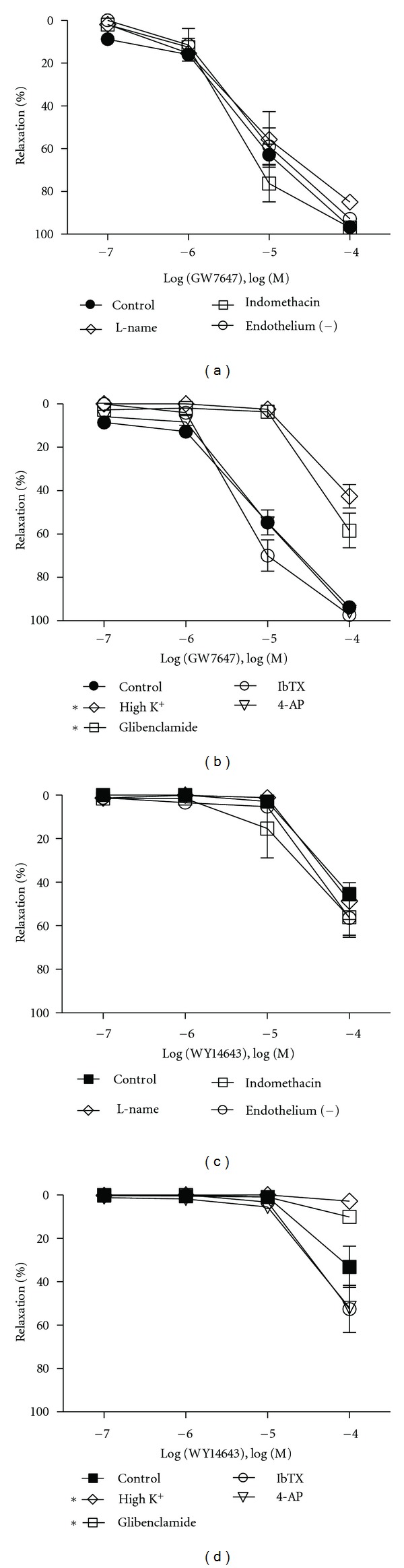
Role of endothelium and K^+^channels in PPAR*α* agonist-mediated relaxations. The effects of nitric oxide synthase, cyclooxygenase, and endothelial denudation on the relaxant response of GW7647 (a) and WY14643 (c) in WT mice aortic rings (*n* = 3–5). L-NAME or indomethacin or endothelial denudation had no effect on GW7647- and WY14643-mediated aortic relaxations. Responses to GW7647 (b) and WY14643 (d) after pretreatment of aortic rings with elevated external KCI (80 mM), 10^−7^ M Iberiotoxin (IbTX), 3 × 10^−3^ M 4-aminopyridine (4-AP), 10^−5^ M glibenclamide (*n* = 3–6). Elevated KCI as well as glibenclamide resulted in attenuation of both GW7647- and WY14643-mediated relaxations. The other K^+^channel inhibitors did not, however, alter the relaxations to PPAR*α* agonists. Data are means ± SEM. *Indicates *P* ≤ 0.05.

**Figure 4:**
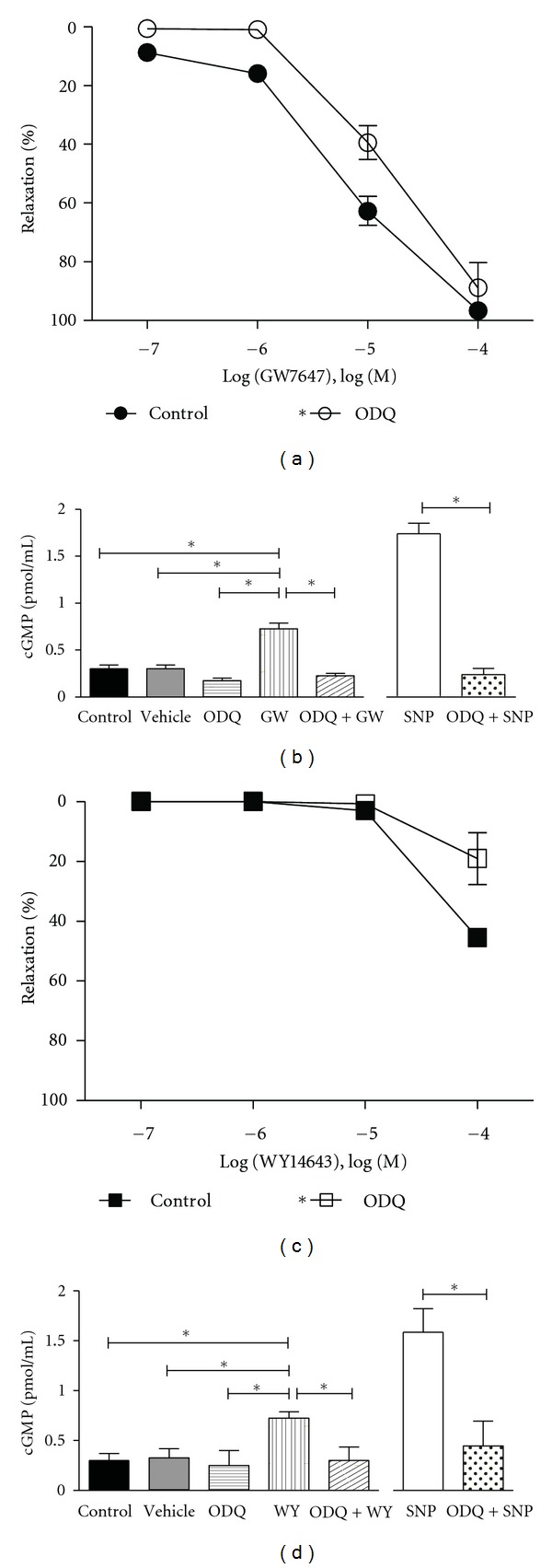
Involvement of sGC in PPAR*α* agonist mediated relaxations. Effect of sGC inhibitor 10^−5^ M ODQ pretreatment on GW7647 (a) and WY14643 (c) mediated relaxations (*n* = 3–5). Preincubation with ODQ resulted in a rightward shift in the GW7647 and WY14643 relaxation curves demonstrating the involvement of sGC. Using an EIA kit, cGMP levels were measured in aortic rings freshly isolated from WT mice under basal and stimulated conditions. (b) 10^−5^ M GW7647 (GW) elevated cGMP content in aortic homogenates 2.4-fold over basal levels, an effect that was inhibitable by pretreatment with the sGC inhibitor 10^−5^ M ODQ (*n* = 4). The NO• donor 10^−6^ M sodium nitroprusside (SNP) elevated cGMP by 5.5-fold in ODQ sensitive manner (*n* = 4). (d) Similar to GW, 10^−4^ M WY14643 (WY) also elevated cGMP by 2.4-fold and ODQ was able to attenuate this response to basal levels (*n* = 4). Compared to untreated control conditions, vehicle treatment (DMSO for GW and WY) did not alter basal cGMP levels. Data are means ± SEM. *Indicates *P* ≤ 0.05.

Relaxation induced by GW7647 and WY14643 in mouse aortic rings was found to be independent of endothelial derived NO• because preincubation of intact aortic rings with the nitric oxide inhibitor L-N^G^-nitroarginine methyl ester (L-NAME, 10^−4^ M) did not impair the response to GW7647 or WY14643. Pretreating aortic rings with indomethacin (10^−5^ M) also did not change the response to GW7647 or WY14643 suggesting that prostacyclin was not involved (Figures 3(a) and 3(c)). Likewise, endothelial denudation of aortic rings also did not modify the GW7647 or WY14643 responses (Figures 3(a) and 3(c)). Potassium (K^+^) channel mediated hyperpolarization of smooth muscle is one possible mechanism of smooth muscle relaxation. Elevating the external K^+^concentration resulted in attenuation of GW7647 (*P* < 0.0001) and WY14643 (*P* = 0.0031) mediated relaxations (Figures 3(b) and 3(d)). Figures 3(b) and 3(d) also show that preincubation of intact aortic rings with Iberiotoxin (IbTX) that blocks large-conductance calcium-activated K^+^ channels (BK_ca_), or 4-aminopyridine (4-AP), which is a blocker of voltage-dependent K^+^ channels (K_*v*_), did not alter the GW7647 or WY14643 responses. In contrast, glibenclamide, which blocks K_ATP_ channels, significantly inhibited GW7647 (*P* < 0.0001) and WY14643 (*P* = 0.0473) induced relaxations (Figures 3(b) and 3(d)). Pretreatment of aortic rings with the sGC inhibitor (ODQ, 10^−5^ M) shifted the GW7647- (*P* = 0.0003) and WY14643- (*P* = 0.0085) mediated relaxation curves to the right (Figures 4(a) and 4(c)). Furthermore, by using aortic homogenates, we found that 10^−5^ M GW7647 elevated cGMP levels by 2.4-fold (*P* = 0.0013) over baseline (Figure 4(b) left). The NO• donor sodium nitroprusside (SNP, 10^−6^ M) likewise increased cGMP levels 5.5-fold (*P* = 0.0006), while 10^−5 ^M ODQ prevented GW7647 (*P* = 0.0058) and SNP (*P* = 0.0010) from elevating cGMP levels (Figure 4(b) right). Similar to GW7647, 10^−4^ M WY14643 also elevated cGMP levels by 2.4-fold (*P* = 0.0041) while ODQ prevented WY14643 (*P* = 0.0109) from elevating cGMP (Figure 4(d)). Thus, it appeared that GW7647 and WY14643 possibly activated sGC, which contributed to the relaxant effects of GW7647 and WY14643.

### 3.4. Mechanism of Dilation Caused by PPAR*α* Agonists in the MCA

**Figure 5:**
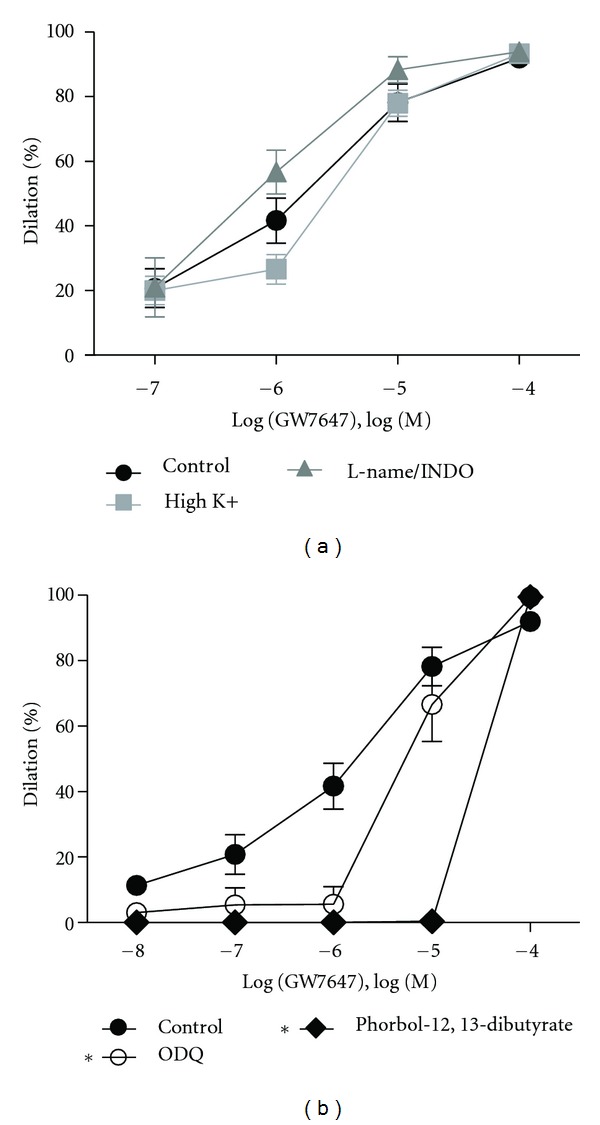
Exploration of the mechanism of PPAR*α* agonist-mediated dilation in MCAs. (a) 10^−5^ M L-name and indomethacin pretreatment did not alter dilation to GW7647 in PE-preconstricted MCAs (*n* = 4). Likewise, in MCAs pretreated with high K^+^, dilation to GW7647 was again unaltered (*n* = 4). (b) In MCAs, pretreatment with phorbol 12,13-dibutyrate, as well as with 10^−5^ M ODQ (*n* = 5) impaired the dilatory response of GW7647 thereby implicating a role for PKC and sGC in the response (*n* = 3). Data are means ± SEM. *Indicates *P* ≤ 0.05.

Since GW7647 and WY14643 relaxant mechanisms were identical in the aorta, and because GW7647 was clearly the more potent dilator, in the demanding MCA studies we focused on the effects of GW7647. In intact MCAs, preincubation with 10^−5^ M L-NAME and indomethacin did not alter dilation to GW7647 (Figure 5(a)), nor did endothelial denudation (not shown). Elevating extracellular K^+^ likewise had no effect on the response to GW7647 (Figure 5(a)) suggesting that GW7647 did not activate K^+^ channels. Preconstricting the MCAs with phorbol 12,13-dibutyrate, which is an activator of PKC, significantly inhibited (*P* < 0.0001) the dilatory response of GW7647 (Figure 5(b)). Similar to the aorta, ODQ also impaired (*P* = 0.0028) dilation to GW7647 (Figure 5(b)) in the MCAs especially at lower GW7647 concentrations, suggesting an additional role of sGC.

## 4. Discussion

In the present study, we examined direct vascular effects of three PPAR*α* agonists. The major findings are as follows. First, PPAR*α* agonists acutely and endothelium independently relaxed mouse conduit arteries and dilated resistance arteries. Second, the response to PPAR*α* agonists was nonreceptor-mediated. Third, in the aorta, the relaxant effects of PPAR*α* agonists appeared to involve K_ATP_ channels and sGC whereas in the MCA, both the sGC and PKC pathways contributed to the response although K^+^channels did not. Use of Ppar*α* ^−/−^ mice demonstrated that these effects were receptor independent. Although PPAR*α* agonists exert anti-inflammatory and antihyperlipidemic effects in vivo [1, 5, 43], our findings suggest that a direct nongenomic vascular smooth muscle relaxant effect may be a contributor to cardiovascular/cerebrovascular protection by PPAR*α* agonists.

Using arteries isolated from wild-type C57/B6 mice, we found that PPAR*α* agonists elicited relaxation of aortic rings, as well as dilation of MCAs. Similar results using gemfibrozil were found in rat tail arteries by Phelps and Peuler (2010), and in rat thoracic aorta by Liu et al. (2012), although in those studies the mechanism of relaxation was not fully explored [36, 37]. As mentioned above, it was recently found that the fibrates, gemfibrozil, fenofibric acid, and bezafibrate also caused relaxation of rat thoracic aorta [37]. Nevertheless, relaxation of rat aortas to these compounds occurred only at high concentrations, and even so was not complete. Similarly, we found that gemfibrozil was without effect until 10^−4^ M; however, we did not pursue higher concentrations because in preliminary studies we observed that arteries did not recover from concentrations greater than 10^−4^ M. In our study, it was interesting to note that the most effective compound, GW7647, also has the greatest pharmacological selectivity for PPAR*α* versus the other PPAR subtypes with an EC_50_ for receptor activation of mouse PPAR*α* of only 1.0 × 10^−9^ M [42]. WY14647, which was a less effective relaxant/dilator than GW7647, has a higher EC_50_ for PPAR*α* activation at 6.3 × 10^−7^ M [42]. Gemfibrozil, which at 10^−4^ M essentially did not act on the aorta, and only weakly dilated the MCAs, has the highest EC_50_ for PPAR*α* receptor activation at 4.5 × 10^−5^ M [42, 44]. Thus, although the relaxant/dilatory potential paralleled PPAR*α* selectivity, this was apparently coincidental because an identical effect was also observed in Ppar*α* ^−/−^ mice. 

In our preparation, the relaxant effects of PPAR*α* agonists were rapid occurring within minutes of addition to the bath. In pancreatic beta-cells, PPAR*α* agonists also have rapid nongenomic effects such as decreasing glucose-induced intracellular calcium signals, and insulin secretion [45]. In addition, Jiménez et al.(2010) also reported nongenomic effects of PPAR*β* agonists on rat aortic relaxations [46]. Agonists for PPAR*β*/*δ*/*γ* were all also found to inhibit platelet activation independent of their genomic effects [47, 48]. Considering that our responses were rapid, and were also observed in Ppar*α* ^−/−^ mice, it was likely that effects were nonspecific, acting instead on other sites. 

In an attempt to discover if the arterial effects of GW7647 and WY14643 were endothelium-dependent, we found that L-NAME and/or indomethacin did not alter the responses in either the aorta or MCA. Thus, NO• and prostacyclin production in endothelium could not have been responsible for the GW7647 and WY14643 mediated responses. Furthermore, endothelial denudation did not alter the response to GW7647 and WY14643 in both the arteries, thereby showing that no endothelial factor, including endothelium-derived hyperpolarization factor could have accounted for the effect. Thus, the acute vascular effects of PPAR*α* agonists we observed in both conduit and resistance arteries were endothelium-independent, targeting instead the smooth muscle.

To explore the role of smooth muscle in PPAR*α* agonist-mediated relaxations, first we examined K^+^ channel involvement in general by elevating the external K^+^concentration. In the aorta, high K^+^inhibited relaxations to GW7647 and WY14643 whereas no effect was observed in the MCA, indicating that the mechanism of action differs by vascular bed. Inhibiting BK_ca_ channels with IbTX, or K_*v*_ channels with 4-AP, did not alter aortic responses to GW7647 or WY14643. The K_ATP_ channel inhibitor glibenclamide, however, was able to inhibit the response. Thus, in the aorta, relaxations caused by PPAR*α* agonists were partially dependent on activation of K_ATP_ channels. 

In vascular smooth muscle, one of the pathways that lead to relaxation involves an increase in intracellular cGMP by sGC. Interestingly, blockade of sGC with ODQ also inhibited responses to PPAR*α* agonists in both arteries. But, in aorta, sGC inhibition was not as consistent as K_ATP_ channel blockade. Relaxations to GW7647 were only moderately reduced by ODQ, although relaxations to WY14643 were basically eliminated. To further explore this finding, in aortic homogenates we found that both GW7647 and WY14643 raised cGMP concentrations to similar levels by activating sGC, although at different concentrations. Just as greater concentrations of WY14643 than GW7647 were required to relax aortas, a similar elevation of cGMP content was achieved at 10^−4^ M WY14643, but only 10^−5^ M GW7647. Thus, our results suggest that in addition to potentially activating K_ATP_ channels, PPAR*α* agonists could also activate these channels via sGC [49]. In the MCA, dilation to GW7647 was also blocked by ODQ, but only at low concentrations of GW7647. This suggests that the dilatory response of GW7647 in the MCA was only partially dependent on the activation of sGC. Together, these results indicate that PPAR*α* agonists appear to activate sGC in conduit and resistance arteries, while in the aorta they also activate K_ATP_ channels. 

Since dilation of MCA to GW7647 was refractory to everything except inhibition of sGC, which was only moderately effective, we sought other targets of GW7647. In cerebral arteries, PKC contributes to myogenic tone [50–52], and we found that pharmacologically activating PKC constricted the mouse MCA. Furthermore, PKC activation largely prevented GW7647 from dilating the MCA suggesting that GW7647 possibly targets this enzyme or an upstream activator of the PKC pathway. While a full exploration of this aspect of the mechanism is beyond the scope of the current investigation, it is unlikely, however, that GW7647 inhibits PKC activation via interactions with other PPAR subtypes. This is because GW7647 is ~200-fold more selective for PPAR*α* than PPAR*γ* or PPAR*δ*. The EC_50_ of GW7647 for PPAR*α* is 0.006 *μ*M, but is a much greater 1.1 *μ*M for PPAR*γ*, and 6.2 *μ*M for PPAR*δ* [42]. While the in vivo importance of PKC in resistance arteries such as the MCA is incompletely understood, it may be that PKC could even become a target for PPAR*α* agonists in conduit arteries. For example, hypertension can lead to the development of PKC dependent basal tone in the aorta [53]. Thus, in certain disease conditions PPAR*α* agonists may affect both the sGC and PKC pathways in MCAs and aortas. 

PPAR*α* agonists also apparently alter vascular function in vivo. For example, the PPAR*α* agonists gemfibrozil [36] and bezafibrate [54] acutely lowered blood pressure in rats. Since PPAR*α* agonists are given to dyslipidemic patients who are also frequently hypertensive [55], direct vasorelaxant effects along with genomic effects may combine to produce cardiovascular protection. Interestingly, chronically administering mice PPAR*α* agonists appears to improve endothelial function. For example, ten days of fenofibrate treatment in mice enhanced endothelium-dependent dilation of mesenteric arterioles as well as relaxation of aortas, possibly by increasing arterial antioxidant capacity, and thus NO• bioavailability [28]. Likewise, fourteen days in vivo treatment with fenofibrate modestly improved endothelium-dependent dilation of the MCA [29]. Although the mechanism of enhanced endothelial function in MCAs was not explored, it was associated with neuroprotection by fenofibrate. Thus, it may be that PPAR*α* agonists evince cardiovascular/cerebrovascular benefits by promoting expression of protective genes via PPAR*α*, and by direct albeit nonreceptor mediated effects on vascular tone. 

In summary, we have demonstrated that PPAR*α* agonists directly and acutely relaxed mouse aortas held under isometric tension, and dilated pressurized MCAs. These responses were nonreceptor mediated, and smooth muscle specific. In aorta, the response depended upon elevations of cGMP by activation of sGC and also activation of K_ATP_ channels. In the MCA, the responses were dependent upon sGC activation and PKC inhibition. Thus, PPAR*α* agonists may protect the cardiovascular system, in part, by directly promoting relaxation of vascular smooth muscle, which has the potential to affect blood pressure by lowering peripheral vascular resistance. This suggests that continued research into cardioprotective PPAR*α* agonists is warranted. Indeed, a class of drugs that are lipid lowering, anti-inflammatory, and that can genomically activate the NO• signaling pathway without the negative side effects of nitrovasodilators [56], as well as directly promote relaxation of vascular smooth muscle could prove very useful.

## 5. Conclusions

PPAR*α* agonists are in wide clinical use and protect the cardiovascular system. Our results demonstrated acute, nonreceptor-mediated effects of PPAR*α* agonists on conduit and cerebral resistance arteries. Protection against cardiovascular disease by PPAR*α* agonists may therefore result from long-term genomic, nonspecific acute effects in the cardiovascular system.
